# The roles of water, sanitation and hygiene in reducing schistosomiasis: a review

**DOI:** 10.1186/s13071-015-0766-9

**Published:** 2015-03-13

**Authors:** Jack ET Grimes, David Croll, Wendy E Harrison, Jürg Utzinger, Matthew C Freeman, Michael R Templeton

**Affiliations:** Department of Civil and Environmental Engineering, Imperial College London, London, SW7 2AZ UK; Department of Epidemiology and Public Health, Swiss Tropical and Public Health Institute, P.O. Box, , CH-4002 Basel, Switzerland; University of Basel, P.O. Box, , CH-4003 Basel, Switzerland; Schistosomiasis Control Initiative, Imperial College London, London, SW7 2AZ UK; Department of Environmental Health, Rollins School of Public Health, Emory University, Atlanta, GA 30322 USA

**Keywords:** Behaviour, Control, Endod, Environment, Hygiene, Sanitation, Schistosomiasis, Snails, Transmission, Water

## Abstract

Schistosomiasis is a disease caused by infection with blood flukes of the genus *Schistosoma*. Transmission of, and exposure to, the parasite result from faecal or urinary contamination of freshwater containing intermediate host snails, and dermal contact with the same water. The World Health Assembly resolution 65.21 from May 2012 urges member states to eliminate schistosomiasis through preventive chemotherapy (i.e. periodic large-scale administration of the antischistosomal drug praziquantel to school-aged children and other high-risk groups), provision of water, sanitation and hygiene (WASH) and snail control. However, control measures focus almost exclusively on preventive chemotherapy, while only few studies made an attempt to determine the impact of upgraded access to safe water, adequate sanitation and good hygiene on schistosome transmission. We recently completed a systematic review and meta-analysis pertaining to WASH and schistosomiasis and found that people with safe water and adequate sanitation have significantly lower odds of a *Schistosoma* infection. Importantly though, the transmission of schistosomiasis is deeply entrenched in social-ecological systems, and hence is governed by setting-specific cultural and environmental factors that determine human behaviour and snail populations. Here, we provide a comprehensive review of the literature, which explores the transmission routes of schistosomes, particularly focussing on how these might be disrupted with WASH-related technologies and human behaviour. Additionally, future research directions in this area are highlighted.

## Background

It is currently estimated that more than 230 million people are infected with schistosomes [1], with an additional 500 million at risk of infection [2]. Three schistosome species comprise the majority of these infections: *Schistosoma haematobium* (occurs mainly in sub-Saharan Africa), *S. japonicum* (distribution restricted to the People’s Republic of China, Indonesia and the Philippines) and *S. mansoni* (mainly in sub-Saharan Africa, Brazil and Caribbean islands). The first species causes urogenital schistosomiasis, and parasite eggs are released in the urine, whilst *S. japonicum* and *S. mansoni* are the causative agents of intestinal schistosomiasis, with parasite eggs released in the faeces [3,4]. Infection occurs when people contact freshwater bodies infested with cercariae released by specific intermediate host snails, which have previously been infected by miracidia released from the eggs mentioned above.

Chronic intestinal schistosomiasis is manifested by debilitating symptoms, such as hepatosplenomegaly (enlargement of the liver and spleen) [3,4]. Urogenital schistosomiasis is associated with significant bladder pathology and an increased risk of developing bladder cancer [5] and thought to exacerbate the transmission of HIV and its progression to AIDS [6]. Schistosomiasis is an aetiological factor of anaemia and malnutrition [7]. Occasionally, parasite eggs enter the central nervous system, causing symptoms such as seizures and focal neurological deficits [3,4,8].

Praziquantel, a safe and efficacious antischistosomal drug, has become the key tool in the global strategy against schistosomiasis [9]. An adult schistosome’s natural lifespan is estimated to be around 5 to 10 years [10], demonstrating the importance of chemotherapy. However, without improvements in environmental conditions, reinfection can occur shortly after treatment, necessitating periodic administration of praziquantel, once every one or two years, depending on prevalence rates [11-13]. In May 2012, the World Health Assembly (WHA) declared schistosomiasis elimination to be feasible in some member states (WHA resolution 65.21), and encouraged water, sanitation and hygiene education (WASH) as components of an integrated control and elimination strategy, on the basis that they should reduce transmission by containing schistosome eggs and reducing human water contact. Soap use related to hygiene may also have a role to play in schistosomiasis control, since soap and endod (a natural soap substitute) are toxic to cercariae, miracidia and specific freshwater snails, suggesting that their use during human water contact may protect from schistosome infection [14,15].

WASH conditions are inadequate in large parts of low- and middle-income countries where schistosomiasis is endemic [3,4,16,17]. Hence, in recent years, the need for multisectoral and integrated approaches to the control of schistosomiasis and other neglected tropical diseases (NTDs) has been emphasised [12,18-35]. In a recent systematic review and meta-analysis of the relationship between safe water, adequate sanitation, good hygiene and schistosomiasis [36], we found that people with safe water had significantly lower odds of a *Schistosoma* infection, as did those with adequate sanitation. However, we did not find any studies comparing odds of infection with soap use during water contact. Moreover, considerable heterogeneities in our meta-analyses suggest that the impact of WASH on schistosomiasis is highly setting-specific, possibly depending on environmental factors such as the location of freshwater bodies and the presence of intermediate host snails, as well as social and cultural factors which govern people’s water contact and contamination behaviour.

Meta-analyses of observational data have the advantage of building large sample sizes through the inclusion of many different studies. However, significant associations between WASH and schistosome infection may result from confounding by socioeconomic status (SES) and other factors. In some settings, everyone has contact with infectious water and SES is unimportant [37,38]. Conversely, in many other settings, people with higher SES have better WASH but are also protected from infection by virtue of having deeper health-related knowledge, better healthcare and access to treatment, and less occupational exposure to infested water [39-41]. Schmidt [42] recently discussed the difficulties inherent in assessing the impact of water and sanitation on disease, and drew attention to the lack of research on the causal pathways through which water and sanitation may impact on health. Similarly, Spear [43] has called for evaluation of environmental pathogen concentrations, in both the prediction and evaluation of risk of infection. Consideration of the schistosome life cycles presents an alternative route of addressing how WASH might impact upon transmission.

In contrast to many other water- and excreta-related diseases where improvements to water supply focus on preventing consumption of contaminated water [44], since schistosomes infect people by passing through intact skin, the success of water supply improvements in preventing schistosome infection depends on the prevention of water contact. Moreover, the parasite stages in the excreta (*Schistosoma* eggs that release miracidia) do not pose a direct threat to humans, being infective only to intermediate host snails, which, some weeks after infection, begin to release cercariae – the stage infective to humans. Therefore, the role of sanitation in schistosomiasis control is to prevent the contamination of freshwater with excreta, rather than to prevent the ingestion of faecal pathogens. Since the parasite stages in the excreta cannot directly infect people, hand washing following defaecation or urination will not affect schistosome transmission – instead the role of soap in schistosomiasis control is to reduce the infectivity of cercariae which might otherwise infect people during contact with freshwater, and perhaps to reduce the infectivity of miracidia, and reduce snail numbers.

Here we review behavioural, biological and experimental studies pertaining to WASH for schistosomiasis control. We also explore potential reasons for the considerable heterogeneities revealed by our previous work on the relationship between WASH on schistosome transmission [36], and review the current state of research regarding WASH for schistosomiasis control, highlighting current gaps in the literature.

### Schistosome life cycles and disruption with upgraded WASH

Schistosomiasis control aims to reduce the propagation of various life cycle stages, and the schistosome life cycles differ from those of other water-related pathogens, with important ramifications for environmental control. Adult schistosomes mostly live as pairs in the perivesical (*S. haematobium*) or the mesenteric (*S. mansoni* and *S. japonicum*) venous plexus of the definitive host – humans and, particularly in the case of *S. japonicum,* other mammals [45]. Female *S. mansoni* and *S. haematobium* worms produce hundreds of eggs per day, while *S. japonicum* females lay thousands per day [46,47]. More than half of these eggs are retained in the host, giving rise to inflammatory reactions, which are the cause of morbidity [3,4,48]. The other eggs are released in the urine or the faeces, depending on the *Schistosoma* species. Eggs that enter freshwater bodies hatch, and each egg releases a miracidium. In the case of a Senegalese endemic community, it was estimated that one stool reaching freshwater might yield around 2,500 *S. mansoni* miracidia [49]. The miracidium is a free-living stage that attempts to infect an intermediate host snail (*Biomphalaria* spp. for *S. mansoni*, *Bulinus* spp. for *S. haematobium* and *Oncomelania* spp. for *S. japonicum*) [3,4].

Inside the snail, the miracidium undergoes asexual reproduction, giving rise to cercariae, which are then released back into the water – around 200 per day in the case of *S. haematobium,* 250*–*600 per day in the case of *S. mansoni* [47], and usually around 15 but occasionally up to around 160 per day in the case of *S. japonicum* [50]*.* Over time, one miracidium may divide into more than 20,000 schistosome cercariae [47], demonstrating the non-linear relationship between water contamination and risk of infection. These cercariae seek out a definitive host and attempt to infect it by penetrating the skin. Inside the definitive host, they develop into schistosomula, then spend 4–6 weeks in the liver, developing further into adult schistosomes [3,4]. Adult schistosomes eventually form male–female pairs and travel to the perivesical or mesenteric venous plexus, completing the cycle.

### Human water contact in relation to safe water supplies

Human contact with cercariae-infested water causes *Schistosoma* infection, so if such water contact could be completely prevented, then transmission of the parasite would stop. However, even if safe water supplies reduce such water contact, they may not completely prevent it. The proportion of water contact that continues with safe water supplies may vary widely between different groups of people and between settings, as a result of cultural, environmental and socioeconomic differences. Furthermore, it is not clear that the amount of water contact is necessarily the limiting factor in schistosome infections, since a host’s immunity and physiology – for example skin thickness – also play a role in preventing infection. If immunity and physiology, rather than the amount of water contact, are the limiting factors in schistosome infections, then water supplies that reduce – but do not completely prevent – water contact, may have little impact on the overall transmission of schistosomiasis.

Schistosomes infect people primarily by penetrating the skin [4], although experiments with human schistosomes in monkeys [51], and *S. bovis* in goats [52], suggest that drinking infested water can also cause infection. Early studies found that cercariae could pass through sand filters [53,54], but they are susceptible to chlorination [55], and flocculation with *Moringa oleifera* [56]. Cercariae are non-feeding, and hence they cannot survive for more than one or two days without infecting a definitive host. Whitfield and colleagues [57], for example, observed that both the survival and the infectivity of *S. mansoni* cercariae begins to decrease after around 10 hours in the water, with very few lasting for longer than 20 hours in the water. Water storage for 24–48 hours before use has therefore long been advocated as a way to prevent schistosome infection – even as far back as 1915 [58,59]. The production of cercariae requires the presence of snails in addition to faecal or urinary contamination, so water from ‘improved’ sources – as defined by the World Health Organization (WHO) and UNICEF joint monitoring programme for water supply and sanitation (JMP) [17] – could reasonably be expected to be schistosome-free. Therefore, water might be considered ‘safe’ in terms of schistosomiasis if it is from a source defined as improved by the JMP, or has not contained an intermediate host snail for at least 48 hours.

While safe water – as defined above – is unlikely to contain cercariae, its provision will often not prevent *all* human contact with infested water. In some settings, activities such as fishing, sand harvesting and car washing account for considerable occupational water contact that safe water supplies would not prevent [60-62]. Similarly, in the People’s Republic of China, groups such as flood relief workers, irrigation workers, canal cleaners and tourists have suffered particularly high exposure to infested water [63,64]. In Brazil, Massara and colleagues [65] found that people who crossed streams were at significantly higher risk of *S. mansoni* infection, and thus inferred that providing water supplies would do little to interrupt transmission.

Quantification of water contact is central to the consideration of how much exposure might be prevented with safe water supplies. Some studies have used the product of area of body surface exposed to the water and duration of exposure [66-69]. Others [70-72] have weighted exposure according to the time of the day, since cercaria concentrations follow diurnal cycles, peaking around midday for *S. mansoni* and *S. haematobium*, and at night for *S. japonicum* [73-75]. Seasonality has also been accounted for since snail numbers, and thus the risk of infection, varies according to changes in temperature, rainfall and irrigation practices [76-80]. Tiglao and Camacho [81] found that activities such as bathing and washing farm animals, which involved little movement in the water, were the strongest predictors of *S. japonicum* infection, suggesting that movement during water contact may be another important determinant of infection.

Laundry, bathing and recreational swimming are often among the activities causing the most exposure to cercaria-infested water, while the collection of water for drinking may be relatively unimportant, often involving the immersion of small areas of body surface, for relatively short durations [60,82-90]. Furthermore, recreational swimming is often engaged in by children, while in many settings laundry is carried out by women, accompanied by their young children who simultaneously are exposed to cercariae and, if infected, contaminate the water with eggs in their urine or faeces [91-98]. The provision of safe and adequate facilities, such as sinks with adequate privacy and drainage for laundry and bathing, and safe areas for recreational swimming, is therefore key to the prevention of *Schistosoma* infections in children. Where such facilities are not available, or safe water is scarce, it may be used for drinking and cooking but laundry and bathing may continue to cause contact with infested water [99,100]. In Nigeria, Akogun [101] found that the ratio of people to wells in four rural communities was significantly associated with the *S. mansoni* and *S. haematobium* prevalences. Data from the Gezira-Managil irrigation scheme in Sudan, and Riche Fond in St. Lucia, showed that as the amount of safe water consumed per capita increased, *S. mansoni* prevalence decreased, until a *per capita* consumption of about 70 l/day, after which it levelled off, apparently due to residual agricultural and recreational water contact that the water supplies could not prevent [102].

‘Comprehensive’ water supplies – those that include facilities such as washing sinks, showers and even swimming pools, have shown success in removing laundry, bathing and recreational swimming from schistosome transmission sites. Pitchford, in a series of studies conducted in South Africa in the late 1960s, investigated the effect of providing swimming pools, water supplies, sanitation, fencing along water bodies and chemotherapy with hycanthone and ambilhar [103-105]. The prevalences of both *S. haematobium* and *S. mansoni* gradually decreased over the following nine years. Jordan and colleagues, in St. Lucia in the 1990s, investigated the provision of water supplies, including swimming pools for recreation, showers and laundry units [106-109]. The intervention area had a significantly lower incidence of *S. mansoni* infection than a comparison area supplied with standpipes only, a result attributed to the continued use of river water for washing clothes there. More recently, Kosinski *et al.* [110,111] investigated the impact of a water recreation area on *S. haematobium* infection in Ghana. A significant reduction in the incidence of infection among local schoolchildren demonstrated the potential of swimming pools to prevent reinfection following preventive chemotherapy.

Elsewhere, though, safe water supplies have not been used, even for those activities to which they seem best suited – and these activities have therefore continued to cause contact with infested river water. The principle underlying reasons appear to be (i) long distances to and (ii) overcrowding at safe water sources such as boreholes and standpipes [112-114]. Other important factors include (iii) a lack of privacy at such sources – which is especially important when considering bathing; (iv) the higher chemical hardness of groundwater, as a result of which more soap is needed for washing; (v) a preference for the flavour of river water; and (vi) the opportunities for social interaction during washing afforded by rivers, in contrast to the case whereby water is collected from a safe source and the washing takes place at home [86,91,115-121]. Additionally, faulty boreholes can leak oil into the water, staining clothes and causing future water contact as people return to washing their clothes in the river [122]. Local superstitions promote contact with infested water in some settings - flowing water is sometimes regarded as cleaner [123] or as ‘life-giving’ [117]. However, other local factors, such as the fear of crocodiles and hippopotamuses, may reduce water contact [124,125], as may the rainy season in some settings – by increasing the availability of water at home, and by rendering river water muddy [126].

**Figure 1:**
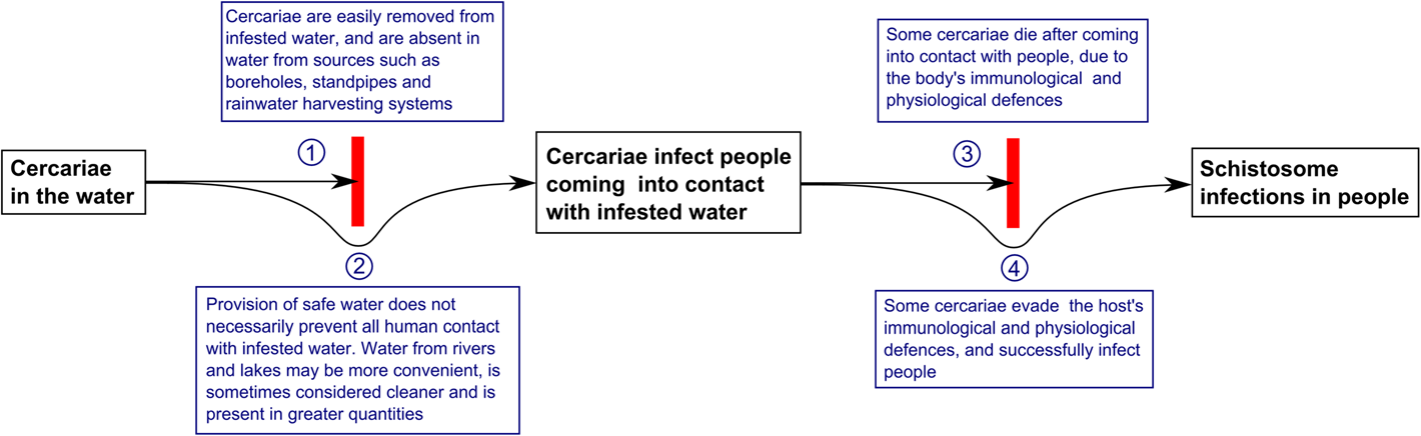
**Flow diagram demonstrating the roles of human water contact, and immunological and physiological factors, in determining schistosome infections.** Point 1 demonstrates that since water from safe sources should be free of cercariae, provision of such water should prevent schistosome infections. However, as shown at point 2, the provision of safe water often does not prevent *all* contact with infested water. Point 3 shows another barrier to schistosome infections, namely the host’s immune system and physiology, which may kill invading cercariae before they can develop into adult schistosomes and cause pathology. Despite the host’s immunological and physiological defences, some cercariae successfully develop into adult worms (point 4). The relative importance of the water contact *versus* immunology and physiology, in preventing schistosome infections, is poorly understood.

In order to cause schistosomiasis, cercariae must both encounter human hosts, as a result of the latter’s contact with infested water, and evade those hosts’ immunological and physiological defences, as is demonstrated in Figure 1. The debate over water contact *versus* immunology and physiology or exposed populations, as the limiting factor in human schistosome infections, remains unresolved and has been summarised by Wilkins *et al.* [127], Anderson [128] and Seto *et al.* [129]. If water contact is the key determinant of infection intensity, then water supply improvements might have an impact on infection intensity roughly in proportion to the amount of water contact that they prevent. However, if immunological and physiological factors such as skin thickness are stronger determinants of infection [130], then high infection rates might persist given reduced but sustained human water contact.

In St. Lucia, each age group’s proportion of all water contacts observed, mirrored their proportion of the total *S. mansoni* infections [131]. Similarly, in Ghana, Dalton and Pole [132] found that the amount of water contact was more important than age in determining *S. haematobium* infection, suggesting that amount of water contact was the most important determinant. The association between amount of water contact and infection has also been found in other studies [70,133-139]. Furthermore, global positioning system (GPS)-assessed water contact frequency in mothers and young children has been found to be associated with *S. mansoni* reinfection at six months after baseline, although not with infection status at baseline [140], and water contact weighted by cercarial risk to be a significant predictor of *S. japonicum* infection even when water contact alone is not [129]. However, in some cases, the amount of time spent near water bodies may be a poor indicator of dermal contact with that water. In Ethiopia, Polderman [141] reported that some women scooping water at a river were able to do so keeping their hands entirely dry, and demonstrated that contact with water carried home from the water body may also pose a risk of infection.

Some studies suggest that age and acquired immunity may be a more important determinant of infection than degree of exposure [61,72,142-148]. The important role of immunity in schistosome infection was emphasised by Woolhouse *et al.* [149], who estimated that fewer than one in a hundred contacts with infested water results in infection with *S. haematobium*, and fewer than one in a thousand results in egg output.

While it is known that schistosome infections occur during contact with infested water, and that some contact with such water may result from inadequate access to safe water supplies, neither the amount of water contact that might be prevented through the provision of safe water, nor the impact of such a reduction in water contact on human infection rates, are well understood. Future research should quantify the impacts of water supplies on water contact arising from various activities, in people of different ages, genders and occupations, and in different settings. It should also continue to use observational studies to investigate the relationship between human water contact and intensity of infection. This knowledge would allow the improved parameterisation of computer models, to determine the expected impacts of various kinds of water supply on schistosome infection in different groups of people. Operational research to monitor infection rates following chemotherapy and provision of different kinds of water supply infrastructure, including elements such as sinks and showers to allow people to use safe water for water-contact activities such as laundry and bathing would provide another method of exploring which forms of water supply can interrupt schistosome transmission, and which other social and behavioural factors affect their effectiveness – as discussed above, this has already been done for a water recreation area [111].

### Sanitation to contain miracidia and prevent snail infections

Schistosomiasis transmission might be expected to be amenable to control through adequate sanitation – defined as infrastructure that contains excreta – since the parasite eggs leave the definitive host in the urine or faeces. By preventing eggs in the excreta from entering freshwater bodies inhabited by intermediate host snails, sanitation should prevent snail infections. A reduction in snail infections, in turn, might be expected to reduce the concentration of cercariae, and hence, the risk of human infection. Miracidia in latrine pits or sewerage systems cannot infect intermediate host snails. However, hygienic bathing and reservoir hosts might provide another source of miracidia in transmission sites, and it is not clear that input of eggs into freshwater is necessarily a limiting factor in schistosome transmission – snail populations and the degree of human water contact may be more important. A given reduction in miracidia does not lead to a proportional reduction in cercariae and human infections, owing to the exponential reproduction of the parasite within the intermediate host snail. More complexity arises from the detrimental effects of schistosome infections on the snails, rendering theoretical predictions of the impact of sanitation on schistosome transmission extremely difficult.

Sanitation systems adequate for schistosomiasis control align with those considered ‘improved’ by the JMP [17], which includes any facility that hygienically separates human waste from human contact. Maldonado and colleagues, in the late 1940s, investigated the survival and hatchability of *S. mansoni* eggs in different environments, as well as the infectivity of miracidia released at different times [150,151]. In a latrine pit, more than 70% of eggs were found to hatch during their first eight hours in water. In a separate experiment, no miracidium was found to survive for longer than nine hours in the water. Kawata and Kruse [152] found similar survival times for *S. mansoni* miracidia in sewage stabilization ponds. Biogas digesters have been found inhospitable to schistosome miracidia, with less than 1% of *S. japonicum* eggs viable after two months [153], a reduction explained as being due to a combination of sedimentation and biochemical inactivation. Thus urine and faeces in adequate sanitation systems are rendered safe in terms of schistosomiasis after relatively short periods of time, while it takes longer for other helminths such as *Ascaris lumbricoides* and *Trichuris trichiura* [154,155]. Sewage sludge would have to run into water bodies containing snails within a few days in order to sustain transmission, and while latrines do sometimes drain directly into water bodies [156], such systems are not considered improved according to JMP guidelines [17].

The presence of adequate sanitation does not necessarily guarantee its use, particularly for urination [5]. Indeed, the bulk of *S. haematobium* eggs reaching freshwater are thought to stem from urination directly into the water, largely by children during bathing and swimming [157]. Open water bodies may be particularly attractive sites for open defaecation and urination, often by men [60,99], for two more reasons: the availability of water for washing following defaecation and the privacy afforded by vegetation, which might be absent elsewhere in the area [158]. In Sudan, privacy was found to be more important than the presence of water for washing [159,160], and this observation was confirmed in subsequent studies in Nigeria and Senegal [161,162]. Such practices vary according to local attitudes: in Brazil and in Ethiopia, people were found to avoid defecating into or near open water bodies which were used for drinking water and bathing [134,141].

Faeces need not enter the water immediately to sustain transmission – those left near water bodies may be washed in during rain or flooding of the river banks, or may be trodden into the river by people or animals [157,158,163]. Eggs of different schistosome species have different longevities – *S. mansoni* can survive for up to about eight days out of the water, while *S. japonicum* eggs may survive for several weeks [151,164].

Even if people *always* use adequate sanitation, it is possible that viable eggs may wash off from the body or from soiled clothing, into the water [141,157,158,165,166]. In Senegal, it was found that hygienic bathing (washing in the river following defaecation) can put significant numbers of schistosome eggs into the water; hygienic bathing by 991 people was found to be equivalent to 12 people defecating directly into the water [49]. Since infection usually occurs outside of the household [71,132], an individual’s risk is therefore determined not just by his or her family’s sanitary practices but by those of the whole community.

In addition to eggs that enter water bodies due to a lack of sanitation coverage or use, more may be provided by reservoir hosts. *S. japonicum* has many animal reservoir hosts, which are understood to contribute significantly to transmission [45,167]. In particular, water buffaloes are often found highly infected [168,169], and in one Chinese village, Wang *et al.* [170] found water buffaloes to account for over 90% of *S. japonicum* egg output. Elsewhere in the People’s Republic of China, combining human chemotherapy with that of bovines has been shown to reduce reinfection in people [171]. Animals may even promote transmission without actually being infected; Wang *et al*. [172] demonstrated that uninfected chickens and dogs may pass viable *S. japonicum* eggs after eating the faeces of an infected host.

**Figure 2:**
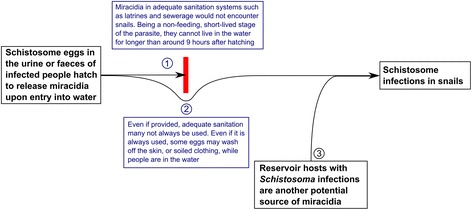
**Flow diagram demonstrating how schistosome transmission may persist despite the use of adequate sanitation.** Schistosome eggs hatch upon entry into freshwater, and release miracida. These miracidia cannot survive for long without infecting an intermediate host, so miracidia in adequate sanitation systems are unlikely to contribute to transmission (point 1). However, it is possible that some eggs may enter freshwater as a result of washing off the bodies or soiled clothing of those infected (point 2). Reservoir hosts provide another potential source of miracidia (point 3).

For *S. mansoni* and *S. haematobium*, however, there is less evidence for important reservoir hosts. Baboons [173-176], chimpanzees [177] and water rats [178-184] have been found naturally infected with *S. mansoni*, and able to pass viable eggs. However, since these were usually around areas of heavy human infection, they are taken as being the result, rather than the cause, of human infection [47]. Primates, pigs, sheep, rodents, monkeys and sea lions have been found naturally infected with *S. haematobium*, but these are thought to be isolated discoveries of limited importance in schistosomiasis transmission [47,185]. Figure 2 shows how the barrier imposed by adequate sanitation may be circumvented by the non-use of the sanitation, eggs washing off the body or clothes of humans, and reservoir hosts.

Macdonald [186], in his early model of the dynamics of schistosomiasis transmission, predicted that even very high levels of sanitation would not have any impact on infection rates. This prediction rested upon the idea that the number of miracidia is not a limiting factor in schistosome transmission – few are needed to maintain snail infections, which give rise to the release of many cercariae, and therefore contamination of water bodies with excreta would have to be reduced to a tiny proportion in order to significantly reduce the force of transmission [186]. Later on, other scientists questioned the general validity of this claim, which was strongly dependent on model parameters and assumptions [187,188]. However, there is consensus that prevention of water contamination would be a less effective control measure than the prevention of human water contact, since, as a result of this reproduction within the snail, many cercariae will continue to be released, even if the number of miracidia in the water is reduced.

**Figure 3:**
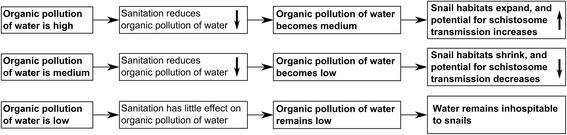
**Flow diagram demonstrating how sanitation may increase or decrease snail numbers, depending on the organic pollution of the water.** By containing excreta, and keeping it away from water bodies, sanitation will reduce organic pollution. However, this may be either detrimental, or under certain circumstances, beneficial to intermediate host snails, that thrive under conditions of mild (but not low or high) organic pollution.

As shown in Figure 3, high organic pollution of a water body may limit the habitats of intermediate host snails, which thrive under mild organic pollution, but are rarely found in areas of heavy pollution [47,189-195]. Sanitation, which acts to reduce such organic pollution, may therefore expand such habitats. With more snails present, the probability of a miracidium finding and infecting a snail is higher [196], and therefore under certain circumstances, sanitation might increase cercaria numbers.

**Figure 4:**
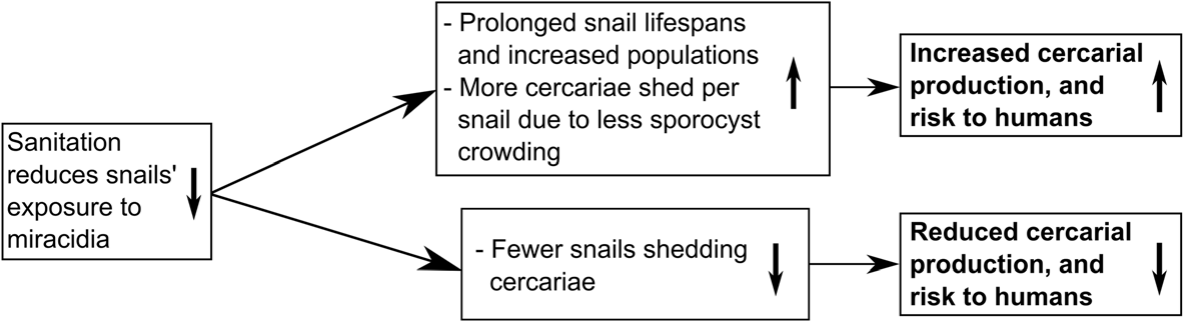
**Flow diagram demonstrating how sanitation may increase or decrease cercarial production depending on the setting.** Reducing snails’ exposure to miracidia may reduce the number of snails infected and shedding cercariae, or under certain circumstances, by reducing the number of miracidia infecting each snail, it may increase snail longevity and cercarial output. Which effect is stronger depends on how frequently snails encounter miracidia, which varies between settings.

The relationship between sanitation and cercarial density is further complicated by the complex interactions between snail schistosome infections, longevity and thus density, and cercarial production. *S. mansoni* and *S. japonicum* infections have been found to increase snail mortality and reduce fecundity [197-199]; effects that are suggested to be more acute in areas of high snail density, due to increased competition for resources [200]. Furthermore, snails infected with many miracidia have been found to yield fewer cercariae than those infected with just one miracidium; an effect referred to as “sporocyst crowding” [197,201]. A computer model developed by Mangal and colleagues [202] predicted that under certain conditions, sanitation might actually exacerbate transmission, as a result of reduced miracidial infection increasing the average snail lifespan and thus the density of snails, all of which nevertheless remain infected by the few miracidia that enter the water even with better sanitation. On the other hand, Sandbach [203] draws attention to the low prevalences of snail infections found even in areas of high human infection rates. From this, the author inferred that schistosomiasis transmission dynamics are much more sensitive to the input of eggs into the water than they are to snail population densities, implying that sanitation might be more effective than snail control. The opposing effects of a reduction in miracidia, on the number of cercariae, is shown in Figure 4.

The JMP measures water and sanitation access in households, and mentions increased access to improved water and sanitation in schools and health centres as key targets [17]. However, there is no mention of improving sanitation in other locations, such as fields and water contact sites, which are perhaps seen as a lower priority. In Zimbabwe, Chimbari *et al.* [122] demonstrated that people working in the fields will not travel long distances to use their household latrines. Raw sewage is often used to fertilise crops, particularly in Asia [204], and this practice has sometimes been found to be associated with a greater risk of schistosome infection [205].

It is well understood that eggs in latrine pits mostly do not contribute to schistosome transmission, but that eggs may also enter the water despite the use of latrines. Research is needed to quantify the reductions in miracidial contamination of water bodies that sanitation may achieve – and computer models should determine whether these might be sufficient to make this part of the life cycle a limiting factor. As with water supplies, operational studies should monitor infection rates following chemotherapy and the improvement of sanitation. For example, randomised controlled trials could test latrine provision in different settings, in particular in fields and as close as practically possible to transmission sites. Reservoir hosts represent a possible source of miracidia that cannot be controlled through sanitation, and studies are needed to quantify the number of *S. mansoni* and *S. haematobium* eggs that they contribute to transmission sites, in order to determine their importance for transmission once human infection rates fall. Moreover, it should be determined whether sanitation provision or reservoir host control might be a more cost-effective method of reducing the input of miracidia into freshwater bodies.

### Soap use during water contact to control cercariae, snails and miracidia

In addition to water supplies and sanitation, one aspect of hygiene – the use of soap or endod (a natural soap substitute) during water contact – may play a role in schistosomiasis control, due to the demonstrated toxicities of soap and endod towards various schistosome life cycle stages. Detergents are understood to be toxic to cercariae of *S. mansoni* [206,207], *S. mattheei* [208] and also to *Biomphalaria glabrata*, the intermediate host snail of *S. mansoni* in Latin America [209]. Okwuosa and Osuala [14] tested the protective effects of different concentrations of washing soaps, on mice immersed in water containing *S. mansoni* cercariae, and found that even at sub-lethal concentrations, these soaps suppressed the infectivity of cercariae.

Many plants have molluscicidal poperties, but endod (*Phytolacca dodecandra*, or soapberry) is of particular interest here since it also has a hygienic use – it is sometimes employed for washing clothes [210]. Its toxicity to snails, and at lower concentrations, to miracidia and cercariae, is the result of a saponin named ‘lemmatoxin’ [15,211-213]. As with soap, sub-lethal doses of endod reduce the infectivity of schistosome larvae.

In view of these findings, the use of soap or endod in the washing of clothes might confer some protection both immediately, by killing or reducing the infectivity of cercariae which would otherwise infect people washing clothes in environmental water bodies, but also in the long term, by killing snails and miracidia, and by reducing the infectivity of miracidia. Indeed, some studies have accounted for soap use, which may explain lower infection rates than would be expected based purely on the amount of water contact [71,214-216]. In Zanzibar, Rudge *et al.* [215] found that washing clothes was not significantly associated with a higher risk of *S. haematobium* infection, possibly due to the protective effects of soap which was widely used in this setting. However, Garba *et al.* [94] found that the use of soap during bathing did not appear to protect infants from schistosomiasis, and suggested that this was due to the long bathing times involved.

Erko *et al.* [217], in Ethiopia, investigated different methods of application of endod to water bodies. In a town using endod soap, the prevalence among males rose slightly (the difference was not statistically significant), while the prevalence among females dropped significantly [217]. This may have been due to women and girls being more likely to have water contact during activities such as washing clothes, which involve the use of soap.

As with water and sanitation, past studies have informed the rationale for schistosomiasis control through soap and endod use during water contact, but only Erko *et al.* [217] have performed a trial to investigate the impact on human infection rates. Quantification of the impacts of such hygiene promotion on snail, miracidium and cercaria populations, would enable researchers to specifically parameterise computer models to determine whether the use of soap or endod can give rise to a limiting factor in schistosome transmission. Such models, in turn, could quantify the impact of specific hygiene promotion on the risk of infection in (i) participants using soap or endod, which might protect them by killing cercaria or otherwise rendering them uninfective and in (ii) other people coming into contact with the water, whose risk of infection might be lowered as a result of reductions in snail and miracidium populations. As with water and sanitation, further intervention studies could test any impact directly, through the assessment of the rapidity of reinfection following chemotherapy. Clearly schistosomiasis control using soap or endod is heavily dependent on human behaviour in addition to the dynamics of schistosome transmission. It would therefore be beneficial for these studies to include social and behavioural components to investigate the acceptability of soap use during water contact in different settings, along with whether health education can increase soap use during human water contact in the long term.

### Summary of evidence regarding the roles of WASH in schistosomiasis control

There are good reasons to believe that improvements in WASH should, in general, reduce the force of schistosomiasis transmission, even if their impacts are highly dependent on the social-ecological context, due to a combination of behavioural, biological, cultural, demographic, ecological, environmental and socioeconomic factors [36,218]. However, the pathways prevented by WASH technologies and human behaviours differ for schistosomiasis as compared to other enteric diseases. Reductions in different parts of the schistosome life cycle (such as contamination of freshwater with miracidia, and human exposure to cercaria-infested water) will only affect schistosome transmission if the part in question is, or becomes, the limiting factor – and which is the limiting factor in schistosome transmission will likely vary from one setting to another.

**Figure 5:**
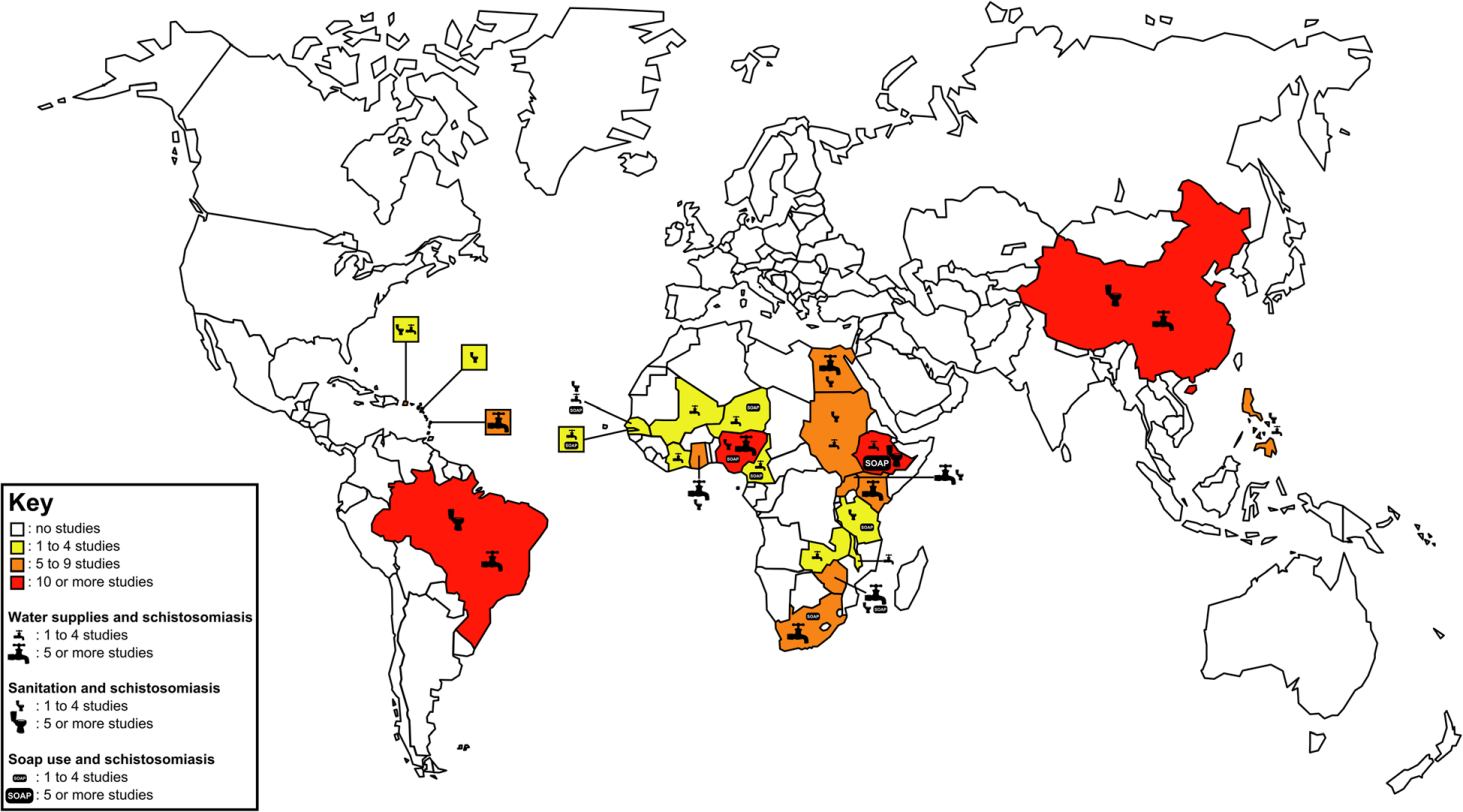
**A world map of the 138 primary field studies cited in this review.** The remaining 81 papers, being reviews, laboratory studies and commentaries, are not displayed on this map. Note that (i) South Sudan and Sudan are shown together since the studies from this region were all conducted before South Sudan became independent in 2011, (ii) Zanzibar is counted as a part of Tanzania, and (iii) studies’ countries were mutually exclusive but their topics of study (i.e. water supplies, sanitation and hygiene) were not.

Qualitative reviews can complement systematic reviews and meta-analyses by exploring heterogeneities in the risk of infection with *Schistosoma* and other causative agents of NTDs, depending on the prevailing social-ecological systems. It is well understood that schistosomiasis transmission occurs focally, in freshwater bodies that are contaminated with human faeces or urine, that are inhabited by specific intermediate host snails and where human water contact patterns occur. However, schistosomiasis control is currently focused on killing the adult worms in the human body through periodic administration of praziquantel, while only token attention is given to the underlying ecology, which includes complex relationships between people’s behaviour, the parasites and intermediate host snails. As shown in Figure 5, many studies across schistosome-endemic parts of the world inform the potential roles that WASH might play in schistosomiasis control and elimination.

## Conclusions

While much is known about the impact of WASH on schistosomiasis, many questions remain. The evidence for specific aspects of water, sanitation and hygiene behaviour reducing schistosome transmission is summarised in Table 1, along with questions that future research might address.

**Table 1:** **Summary of the key points regarding water, sanitation and hygiene for schistosomiasis control, and suggested directions for future research**

**Domain**	**Summary points**	**Open research questions**
Water	• Water from safe supplies is usually schistosome-free, and hence it may play an important role in reducing exposure	• Do communities provided with safe water supplies experience slower reinfection (lower intensities of infection at set points in time following preventive chemotherapy)?
	• People’s motivations for water contact are highly context-dependent, and water supplies that do not account for local attitudes and practices cannot be expected to consistently reduce water contact	• How do different types of water supply infrastructure (e.g. household and community supplies, with and without sinks and showers) affect the amount of exposure to cercaria-infested water in different groups of people (preschool-aged children, school-aged children, adults, males, females and people in communities of different religions and engaged in different forms of water contact)?
	• Where possible, water supplies should incorporate additional infrastructure such as sinks and showers, to prevent as much water contact as possible	• How do reductions in water contact affect the intensity of schistosome infection in different groups of people (preschool-aged children, school-aged children, adults, males, females, those from or not from endemic communities)?
	• The relationships between people’s access to safe water supplies and their degree of water contact, and between their degree of water contact and their intensity of infection, are not well understood	
Sanitation	• Eggs in latrine pits cannot sustain schistosome transmission, but eggs may still enter the water despite the use of adequate sanitation	• Do communities provided with adequate sanitation experience slower reinfection (lower intensities of infection at set points in time following preventive chemotherapy)?
	• High levels of organic pollution, and infection with schistosome sporocysts, are detrimental to intermediate host snails, and therefore the impact on cercaria populations, of a reduction in miracidial contamination, is very difficult to predict	• Is sanitation in fields and near transmission sites effective at reducing the number of miracidia at those transmission sites?
	• By contaminating freshwater bodies with schistosome eggs in their faeces and urine, reservoir hosts play an important role in *S. japonicum* transmission, and may also play a role in *S. mansoni* and *S. haematobium* transmission	• How does the number of snails affect the relationship between the numbers of miracidia and cercariae at transmission sites?
		• What role do reservoir hosts play in the transmission of *S. mansoni* and *S. haematobium*?
Hygiene	• Soap and endod are toxic to miracidia, cercariae and intermediate host snails – they may therefore reduce risk of infection in the short term, by killing and reducing the infectivity of cercariae, and in the long term, by killing snails and miracidia, and thus reducing cercaria populations	• Does sustained soap use during water contact slow reinfection (lower intensities of infection at set points in time following preventive chemotherapy)?
	• Very few studies have compared human use of soap or endod during water contact, with subsequent schistosome infections	• What impact does sustained soap use have on snail, miracidium and cercaria populations?
		• Does protection from infection arising from soap use extend to people not using the soap, by virtue of the impacts on snail populations and miracidial infections?
		• How might soap use during human water contact be best promoted?

The ability of water supplies to prevent water contact depends on the local activities involving water contact, convenience, the chemical composition of water and local beliefs and superstitions. Although schistosome infection certainly occurs during water contact, it is difficult to predict the impact of a reduction in water contact on infection, due to the effects of age-acquired immunity.

Access to, and use of, adequate sanitation will catch most *Schistosoma* eggs and prevent miracidia from infecting intermediate host snails. However, sustained transmission requires only a few eggs to enter freshwater, and these do so without people defaecating or urinating into the water. Organic pollution of water bodies, and schistosome infections, can be detrimental to snails, and therefore under certain circumstances, sanitation may exacerbate the transmission of schistosomiasis. Reductions in the input of eggs into freshwater may have no impact if this is not a limiting factor in overall transmission.

The use of soap, detergent and endod during water contact appears to confer some protection from infection, depending on the duration of water contact. However, little is known about the quantitative impact on risk of infection among people engaging in such water contact.

Preventive chemotherapy using the existing education system is inexpensive, by some estimates, less than US$ 0.50 per child per year [219]. However, this results from the economies of scale, which allow for the treatment of many children in one deworming campaign. As countries push towards elimination of schistosomiasis, infection rates will fall, and the cost per person in need of treatment (i.e. those who are infected) will rise. Suitable improvements in WASH may then be particularly beneficial in tackling refractory transmission foci by preventing reinfection following chemotherapy. Such WASH interventions should take account of the many local factors and social-economic contexts that will determine their effectiveness.

## Abbreviations

AIDS: Acquired immune deficiency syndrome
GPS: Global Positioning System
HIV: Human immunodeficiency virus
JMP: The WHO and UNICEF joint monitoring programme for water supply and sanitation
NTDs: Neglected tropical diseases
SES: Socioeconomic status
UNICEF: the United Nations Children’s Fund
WASH: Water, sanitation and hygiene
WHA: World Health Assembly
WHO: World Health Organization
